# Teaching Hygiene for Better Parenthood

**Published:** 1914-01

**Authors:** Thomas D. Wood

**Affiliations:** New York, Professor of Physical Education in Columbia University and College Physician, Teachers’ College


### Teaching Hygiene for Better Parenthood.

BY THOMAS D. WOOD, A. M., M. D., NEW YORK.
Professor of Physical Education in Columbia University and College Physician, Teachers' College.

The teaching of hygiene in a thorough and comprehensive manner should serve many important purposes in the education of young people—in helping to perapre them for the manifold phases of responsibility which life may bring to them. Hygiene instruction, however, can further no more important interest; in fact, none so important as that which relates to the supreme privilege and obligation of life—namely, parenthood. And this obligation, impending even far off beyond the years in the life of the growing child, should be recognized by parent, by teacher and by all the adult guides of the young in a very practical way as a potential responsibility for which human beings are to be trained in a far sighted and effective manner.

We are fond of quoting Oliver Wendell Holmes, philosopher, wise physician and beloved author, as saying that the education of the child should begin two or three generations before he is born. Did he not anticipate in this significant paragraph the present day conception of the principles underlying our ideas of eugenics, of education relating to the more direct and indirect obligations belonging to parenthood?

The child is the most valuable object in the human world.

Biologically speaking, the only reason for the existence of the adult of the species is the reproduction and care of the young. The most important business on earth is the bearing and rearing of children. And it is of all forms of business, in relation to its value to mankind, the most inadequately understood and the least well conducted. This is due in part to two causes:

1. Men are most interested in, and devoted to, the enterprises which affect themselves, their own generation.

2. For the most part they do best the task which appeal directly to the senses or whose results are quickly measurable in terms of money and in the immediate returns which money will buy.

A high quality of parenthood, however, involves many essential ingredients and the most important of them lie well outside of the factors generally considered indispensable for the ordinary and most of the unusual pursuits of mankind, unless the rather sweeping assertion is made that intelligence and common sense are sufficient for all human occupations.

Some of the comparatively rare ingredients which seem desirable for better parenthood may be briefly summarized as follows:

1. For practical eugenics it is essential that the romantic, the affectional basis of marriage should be preserved, but the sentimental and emotional element should be supported and guided by intelligent appreciation of all the factors necessary for parenthood which will protect the biological values, as these are as important in the human species as in any other.

This view of life, in which the interests of the future determine present conduct, will modify the influences and attractive forces which draw men and women together for marriage and the institution of the family. The dominance of this ideal will, unconsciously even, in the minds of those properly trained, tend to make those qualities, attributes and characteristics in each most attractive and desirable to the other which are most important in men and women, in father and mother, for the welfare of the future.

In the mating of men and women, money, social position, worldly expediency, the conventional and fictitious values so influential in these days, will count for much less, while organic health and efficiency, character, unselfish devotion to high ideals, to the great world interests will count for far more. In this obedience to ideals so far sighted, romantic love will not be lost in any way, as some seem to fear. Men and women will not choose one another in cold blood simply because intelligence and reason point the way, but human sentiment and every romantic quality will be enhanced when permanent and future interests are furthered by a saner and finer human choice. Marriage in many of its most important aspects cannot be controlled by law (except in the case of the dependent classes) or by a hard impersonal science, but it may be controlled to immense human advantage through the ennobled and rationalized sentiments of men and women trained from childhood to justly estimate, almost instinctively and automatically, the vital issues of life.

2. Parenthood requires that the child, the young of the species, shall not only be provided with the conditions for physical growth, health and strength; not only with the habits of industry and skill necessary for the maintenance of life and for acquirement of material resources conditioning intellectual and esthetic satisfaction, but also with the standards of thought and conduct; with the controlling motives for action which will constitute the best preliminary training during infancy, childhood and youth for potential parenthood and all that it includes.

When human beings and families rationally subordinate their own interests as perfectly to the welfare of future generations as do animals under the control of instinct the world will have a more enduring type of family life, a more perfect type of parentcraft, than exists at present. This can only be accomplished by the development of controlling ideals which are supported not only by reason and intelligence but by ethical impulse and religious motive. This larger altruism which protects the permanent interests of the future against the more temporary values of the present must be of the heart as much as of the head.

3. Parenthood is the provision for the perpetuation of the species—of the race. The life of the animal is controlled by instinct in the interests of the generations yet unborn.

In the scale of relative values the child is more important than the parent but future generations are again more important than the child. Under our present conditions even when parents are devoted to their children, the child is too often considered as the end of their affectionate care, is frequently more or less spoiled and the true process of human progress is interrupted. Children cannot receive too much thought and care if these are wisely directed, but they can and often do have too much misdirected attention. How often the child is subordinated to the parents' pleasure and pride! He must show off his accomplishments before friends and strangers. He is perhaps over-stimulated with excitement when he should be simply vegetating. He or she is often made the burden of "conspicuous waste"; to display the wealth and indicate the social position of his or her parents, in wearing garments and decorations which interfere with the most wholesome life and activities. Often again the child is overfed or rather given what he pleads for, whether good for him or not, and this may be while he is really suffering from deficient nutrition. Frequently the child is overworked or at least forced beyond heathful limits in school; drawn too early into social life; made to conform to conventional standards in education and society and this either to gratify his own precocious desires or to satisfy the irrational ambition of teachers and parents. All of these phases of developing life are but means to a greater end in the future even of the individual. The child should not be trained for mere personal attainment but for maturity, with reference to the power he will exert later, the influence which he will have in his own life and through this on those who come after.

The home should be considered the place where are to be developed and conveyed the precious qualities which are so vital to the continuity of the race and the progress of human society and civilization.

A higher type of parenthood will possess in itself and develop in the child a long distance view, a projected vision, an imagination which will conserve and foster future values more successfully. How far can the teaching of hygiene contribute to better parenthood? Very little, if it involves simply informative instruction of the traditional type, the imparting of facts relating to reproduction, to heredity, to parenthood. On the other hand, the teaching of hygiene may contribute much if it be a broad and comprehensive hygiene, taking account of the pyschic, social and moral as well as the physical nature of the young; striving to inculcate habits of thought and action sufficient for the present and adequate for the future needs of the child; to inspire ideals and establish standards of life which shall provide for racial and future as well as present and personal needs and obligations.

The phase of human teaching, which is, last of all and most reluctantly, being recognized as essential, appropriate and possible for education and for hygiene; for life preparation of the human individual, is that which relates to the perpetuation of life, to the reproduction of the species, to the thought and conduct which is directly and indirectly associated with this phase, and responsibility of life.

In an analysis of reasons for delay in the development of this department of education, the following suggest themselves:

1. It has been assumed, until recently, by most people, and is still believed by many, that an innocence dependent upon comparative ignorance of reproduction was desirable up to maturity.

This, in part, was due to the general belief that the reproductive instinct and its expression and influence in human life was, in the nature of things, not adapted to rational understanding, organized knowledge and informative teaching. In fact, much of the attempted teaching in this field has consisted in the effort to discourage or suppress inquiry or thought on subjects relating to sex, rather than to encourage suitable, wholesome understanding and control of knowledge and impulses.

2. There has been lacking a definite body of knowledge and opinion regarding the biological, hygienic, psychological, sociological and ethical aspects of sex sufficient for satisfactory education in this field.

Upon no subject of vital importance to the welfare of young people are parents, teachers, physicians and adults in positions of responsibility, so ignorant and lacking in unanimity of knowledge and opinion as with regard to sex in its direct and indirect bearings on the life of the young.

3. The atmosphere of human life, in respect to sex and reproduction has been so often clouded; the stream of human thought, of memory and conduct has been, and is so often, made turbid by various degrees of error, that many really good people fail to fulfil their obligations of instruction and guidance of their own children or of the children of others. Again, not a few parents and teachers, through lack of appropriate training in youth, or because of temperamental limitations, are so constituted that it seems almost impossible for them to teach and guide with frankness and tact their own children.

For the protection and help of their own children, and of young people generally, from error directly or indirectly related to sex, they depend upon the carrying over of facts and principles of science and morals in a way which will insure sufficient wisdom and safety for the young at various ages.

Human experience has abundantly proven that dependence upon such an indirect and round-about process of reasoning, and of guidance for human conduct is illogical and ineffective.

Certain positive reasons present themselves in support of the proposition that: Appropriate education with reference to reproduction and parenthood, is vitally necessary in the development of every young person.

1. The human individual can find himself in relation to knowledge, impulse and responsibilities of sex, at earlier or later stages, with reference to himself or others, only by instruction and influence of those who shall be adequate to supply the need at each stage of experience.

Instinct and intuition are not sufficient for thought, judgment and conduct, which will be satisfactory in this sphere. While conscientious application of general, moral and religious principles to the sex life may save many from flagrant error, still this form of protection is very uncertain, and altogether insufficient for the more positive needs of human life.

2. For the complete development of mind, soul, character, and personality, each child absolutely needs, and hence is entitled to, a normally growing body of knowledge of the great facts and principles of reproduction; of the perpetuation of life; which is involved in the individual increase of consciousness and is related to the developing sense of personal, social, parental, and racial obligation. Parkinson, in his illuminating article on sex education in the January number of the *Educational Review*, has made a valuable contribution to the literature in this field with his clear and stimulating exposition of the beneficial effects of the right kind of sex education in the gradual upbuilding in the young of mind and personality.

3. The third striking reason for sex instruction is to safeguard the young, so far as teaching may accomplish this, against the errors and disasters which come through the violations of the laws of sex hygiene.

The prevention of sex perversion, immorality, and disease, while important as an argument for sex education, and as a motive for sexual morality, has often been made too prominent in the discussion of this subject. The prevalence, character and significance of venereal diseases in the world involve facts of awesome import in relation to the health and morality of the individual, family, community and nation. With these vital problems, all types of responsive agencies must seriously and constructively deal.

The fear of disease, however, is not the highest type of incentive to decent and fine living. It should be employed with all force for those who cannot be effectively appealed to on a higher plane, but the finer considerations and motives should be employed first, and to the full measure whenever there is a reasonable chance for them.

If we assume that sex instruction of the right kind is necessary for every child, then the question arises: When, how and by whom shall such instruction be given?

Without attempt at comprehensive and logical development of this phase of the subject, certain opinions are presented in somewhat categorical fashion for consideration.

The most natural and logical teacher of sex and parenthood to the child is the parent. For the parent, wise and sufficient to opportunity and needs, this may be even more a privilege than a duty. Parents should be urged and helped in every possible way to fulfil, as far as may be possible, their obligation in this field of teaching to their own children.

If parents fail in this duty to their children; if they cannot, or will not, or for any reason whatsoever, do not help their own sons and daughters in this essential aspect of preparation for life, then it is most important that help should be given by person or persons old enough, wise enough, and situated in such a relation to the child that, in view of all the circumstances, such instruction, such guidance, such help, may reasonably and suitably be given. Shall it be admitted, that, in a civilized nation, in a Christian country, in an intelligent community, there may exist a single child who will fail to receive the needed teaching related to the wonderful, the essential truths and principles, the laws of sex, of reproduction?

It is certain that multitudes of children are growing up without any suitable teaching in this field. A very small percentage of the children of this generation are getting, or will get, any adequate instruction and guidance in this department of life.

The children of the rich are, save for certain conventional and temporary elements of protection, no better off than the children of the poor. Orphans sometimes are more helped than many children surrounded in parental homes by luxury and negligence.

It is not proposed here to discuss the problem of classroom or group instruction with reference to direct human sex teaching in the public school beyond the expression of the writer's conviction that such instruction cannot be given until: (a) Enlightened public opinion recognizes sufficiently the necessity for such instruction, and exhibits confidence in the ability of the teachers to give the instruction needed; (b) Teachers are intelligent, wise and tactful enough to give such instruction and guidance successfully. Comparatively few teachers today are capable of meeting the obligations involved in sex education.

Such instruction should be given in universities, in colleges, in normal, and in private schools. It is very important that parents, teachers, physicians, nurses, social workers and all serious adults should in all possible ways be informed upon this subject, and make themselves capable of advising and guiding young people individually, and thus the way may gradually be prepared for the more complete and satisfactory teaching in this field which must come in the not distant future.

With reference to the individual teaching of the child wherever and whenever this may be sensibly possible, some fundamental considerations seem important.

The average normal or typical child up to the beginning of adolescence, at least, is unconscious of sex feeling or impulse. The curiosity of such a child regarding reproduction or sex matters represents the same kind of natural intellectual inquiry which the child might feel or express regarding any interesting phenomena, unless by the unfortunate attitude of elders and companions, the child is made conscious of some peculiar quality or interest attaching to this class of subjects.

There are, of course, some children, who, before they reach their teens, for one reason or another, have become precocious in relation to sex feeling or habit. These should be helped as wisely as possible according to individual needs. It is most important, however, that the simple, natural, and relatively unconscious attitude of the typical child should be preserved, not by withholding knowledge, but by giving all the instruction needed in the moast satisfactory manner.

The child, up to adolescence, will be best taught first by the carrying over of the applications from Nature study, and from the study of life in general, and, second, by the satisfying answers to the freely asked questions of the child as they occur. The possible exceptions to the above might be the simple directions to be given to the child regarding the routine care of the body when the age of self-care is reached.

For the young child, however, the problem is more psychologic than hygienic or moral, and no problem in education requires keener psychologic insight, or finer pedagogic skill than to know how the individual child should have the simple sex questions answered according to nature, temperament, stage of mental development and the rest. It is better not to put any of the sex books into the hands of a child, but the parent and the teacher should be familiar with the best literature in this line, and be able to give the instruction in a direct and satisfactory way.

The basis of successful sex teaching is companionship and confidence between the child and the parents, or the person who stands *in loco parentis* in relation to this phase of instruction. The child should be most inclined to ask for information where this may most safely and wisely be given.

This need of the child for wise and tactful guidance exists up to maturity, and complete education involves for each young person the help of a perfectly qualified and sufficiently interested person during the years of growth, and groping through the mazes of human development.

When class or group instruction is given to adolescents of either sex, it is most important that the primary emphasis should not be given to sexual anatomy or physiology, which may be unnecessary or confusing, or for some undesirably suggestive, but rather to the presentation of facts and values, biologic, hygienic, sociologic, ethical and economic, which will inspire youth to wholesome thought and conduct and warn them against error and harm to themselves or others. The welfare of those who are now, or may be later dependent upon them, will restrain many young people from thoughtless or injurious conduct when consideration for their own safety would not act as an effective deterrent. More important than the knowledge of sex hygiene, then, are the motives, which at different stages of development, and for different types of young people, will control thought and action as effectively as possible.

In this connection may be appreciated the significance of title of Dr. Cabot's paper on Sex Education, which he calls "The Consecration of the Affections." The devotion of a child to sympathetic parent or friend; the devotion of youth to a human ideal; the devotion of a young man to the imagined or actual woman whom he hopes to marry will often be the most compelling influence to hold the individual to a worthy standard of living.—*Medical Review of Reviews.*

